# The Role of NLRP1, NLRP3, and AIM2 Inflammasomes in Psoriasis: Review

**DOI:** 10.3390/ijms22115898

**Published:** 2021-05-31

**Authors:** Magdalena Ciążyńska, Irmina Olejniczak-Staruch, Dorota Sobolewska-Sztychny, Joanna Narbutt, Małgorzata Skibińska, Aleksandra Lesiak

**Affiliations:** 1 Nicolaus Copernicus Multidisciplinary Centre for Oncology and Traumatology, Department of Proliferative Diseases, 93-513 Lodz, Poland; 2 Department of Dermatology, Pediatric Dermatology and Dermatological Oncology, Medical University of Lodz, 91-347 Lodz, Poland; karolkc@gmail.com (I.O.-S.); wojciech.niemko@wp.pl (D.S.-S.); joanka.narbu@wp.pl (J.N.); skibka.malgo@wp.pl (M.S.); leska.leska.ola@wp.pl (A.L.)

**Keywords:** psoriasis, inflammasomes, pathogenesis, NLRP1, NLRP3, AIM2

## Abstract

Inflammasomes are high-molecular-weight protein complexes that may cleave the two main proinflammatory cytokines, pro-interleukin-1β and pro-interleukin-18, into active forms, and contribute to psoriasis. Despite recent advances made in the pathogenesis of psoriasis, mainly studied as an autoimmune condition, activation of immune response triggers of psoriasis is still not completely understood. Recently, focus was placed on the role of inflammasomes in the pathogenesis of psoriasis. Multiple types of inhibitors and activators of various inflammasomes, inflammasome-related genes, and genetic susceptibility loci were recognized in psoriasis. In this systemic review, we collect recent and comprehensive evidence from the inflammasomes, NLRP1, NLRP3, and AIM2, in pathogenesis of psoriasis.

## 1. Introduction

Psoriasis is a chronic inflammatory skin disease that affects nearly 3% of the global population. It is characterized by relapsing, red, scaly plaques and papules [1,2]; it may affect any part of the skin, but mostly occurs on the elbows, knees, and the scalp. Approximately 30% of patients with psoriasis develop seronegative psoriatic arthritis (PsA), usually manifesting as asymmetrical arthritis, which may involve entheses, joints, or even the spine [3]. In addition to psoriatic arthritis, psoriasis is increasingly recognized as a multisystemic disease with metabolic syndrome, obesity, inflammatory bowel disease, cardiovascular disease, and depression (being the most common comorbidities) [4].

Psoriasis is a complex disease in which keratinocytes, immune cells, and other skin cells, including endothelial cells, interact dynamically [5]. Aberrant proliferation and keratinocytes differentiation lead to epidermal hyperplasia and excessive dermal infiltration by numerous immune cells, including dendritic cells (DCs) and T cells. The increased dermal capillary density and excessive production of several inflammatory chemokines and cytokines are the most important features of psoriasis [6]. The interaction between infiltrating immune cells and the keratinocytes observed in the disease is key to the development of an inflammatory response that is primarily mediated by interleukin (IL)-17 [7]. Although, IL-17, IL-23, and tumor necrosis factor (TNF) are recognized as main cytokines involved in the development of psoriasis, more attention is paid to the role of the activation of the multi-protein inflammasome complex in the pathogenesis of psoriasis, which in turn is associated with increased production of IL-1β and IL-18 [8,9,10].

Inflammasomes consist of a group of cytosol protein complexes that play a crucial role in the innate immune response, contributing to the host defense against pathogens and repair processes upon the induction of inflammation [11]. Activation and assembly of the inflammasome complex is mediated by damage-related molecular patterns (DAMP) or by the unrestrained release of pathogen-associated molecular patterns (PAMPs) through specific pattern recognition receptors (PRRs) [12]. Several types of inflammasomes are recognized, depending on the PRR type that is involved, including nucleotide-like receptors (NLR), RIG-like receptors absent in melanoma-like receptors 2 (AIM2), toll-like receptors (TLR), and pyrene. Assembly of the inflammasome components involves recognition inflammasome receptor interaction with the apoptosis-associated speck-like protein, containing a caspase recruitment domain (ASC) and effector protein pro-caspase-1, -5, or -11 [13,14], which leads to the activation of the effector caspase through proteolytic cleavage. In the next step, the activated caspase-1 cleaves pro-IL-18 and pro-IL-1β, producing their active forms. It was reported that increasing IL-18 secretion promotes the maintenance and development of Th17 cells, which are broadly associated with autoimmune inflammatory diseases, such as psoriasis [15]. Moreover, psoriatic skin shows elevated caspase-1 and -5 activities [16].

Earlier studies also showed an increase in the level of IL-18 in psoriatic lesions, correlating meaningfully with the time of the disease and clinical severity [17,18]. Proinflammatory cytokines, secreted in large amounts, activate effector cells, including keratinocytes, macrophages, and neutrophils, which then induce an inflammatory response in damaged tissue.

Therefore, abnormal and chronic activation of inflammasome complexes underlie the pathology of psoriasis due to the increased levels of pro-inflammatory cytokines. Previous studies confirmed the role of genetic components associated with the inflammasome in psoriasis susceptibility [19]. However, the accurate role of inflammasomes in psoriatic arthritis and psoriasis is still mainly undefined.

This extensive systematic review presents the contributions of inflammasomes and immune cells to the pathogenesis of psoriatic arthritis and psoriasis.

**Figure 1:**
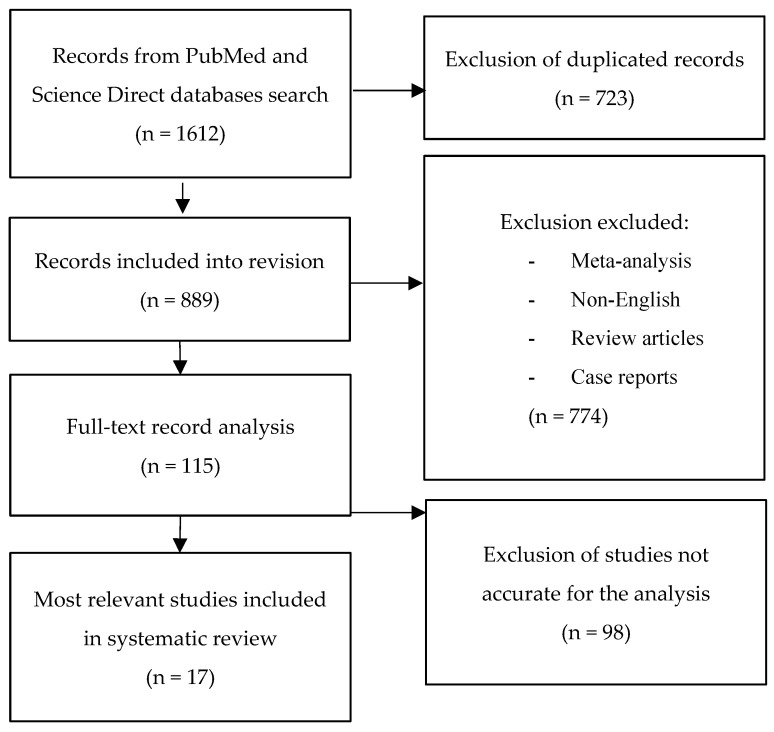
A graphical diagram of the selection of the literature data for the review.

We conducted a review of the literature, investigating the role of inflammasomes and immune cells in the pathogenesis of psoriasis, using a systematic electronic literature search of the PubMed and Science Direct databases. The databases were searched using the terms “inflammasome”, “NLRP1”, “NLRP3” or “AIM2” and “psoriasis” or “psoriatic arthritis”. The search yielded 1612 citations. They were screened and 723 duplicate articles were removed. No exclusion criteria were defined. Using the selection criteria, 889 papers were reviewed by title and abstract and 774 were excluded. A further 115 studies were identified for full-text assessment and 98 papers were excluded. All articles, published until 1 March, 2021, were considered. Seventeen articles qualified for quality evaluation. They were positively assessed and included in this systematic review. The pertinence of each article was evaluated with an attentive and critical view regarding the type and content of the article and the impact factor of the publishing journal. Off-topic publications were eliminated. The study selection scheme is presented in Figure 1. The characteristics of the included studies are summarized in Table 1.

## 2. The NLRP3 Inflammasome (in Psoriasis)

The nucleotide-binding domain leucine-rich repeat (NLR) and pyrin domain containing receptor 3 (NLRP3) inflammasome is the best characterized inflammasome complex, which is composed of NLRs, an apoptosis-associated speck-like protein containing a caspase recruitment domain (ASC), and caspase-1 [33]. NLRP3 may be described by an indirect manner, by a wide range of extracellular inflammatory stimuli, including bacteria, viruses, and other pathogen-associated molecular patterns (PAMPs) and damage-associated molecular patterns (DAMPs). Nevertheless, the molecular mechanisms triggering NLRP3 activation are still not well defined. The protein complexes are activated by various stress factors, including K+ efflux, intracellular Ca_2_^+^ levels, extracellular ATP, mitochondrial dysfunction, reactive oxygen species (ROS), and lysosomal rupture (Table 1).

NLRP3 activation triggers caspase-1 that splices pro-IL-1β and pro- IL-18 to produce active forms of IL-18 and IL-1β [8,34]. Moreover, recent studies have found that NLRP3 normalizes the splicing of gasdermin D (GSDMD) by activating caspase-1 into two fragments (the C and N domains). Then, the N-terminal fragment (GSDMD-N) gathers and forms pores on plasma membrane to induce pyroptosis. Thus, it is suggested that GSDMD is also a main protein component of NLRP3. Furthermore, caspase-11 directly endorses pyroptosis through cleavage of the pore-forming GSDMD and indices an activation of the canonical NLRP3 for cytokine release [21,31].

Abnormally activated immune response is key to psoriasis development and, therefore, in psoriasis, the role of NLRP3 inflammasome activation received extensive curiosity and appreciation. It is confirmed that the NLRP3 inflammasome formation may contribute to psoriasis inflammatory response. In the psoriasis samples, the expression of NLRP3 was four times higher than the expression of NLRP3 in normal skin biopsy samples. Furthermore, results by Su et al. indicate that IL-1β expression levels are higher in comparison to normal skin biopsy samples. Compared to the expression of IL-1β in normal skin biopsy specimens, the expression of IL-1β was approximately 3–4 times higher. Moreover, the expression of caspase-1 was highly increased in psoriasis samples. Caspase-1 gene expression was 2–3 times higher than in normal skin biopsy specimens [22].

Similar observations were found a year earlier in an investigational mouse model of imiquimod-induced psoriasis-like dermatitis. IMQ skin samples presented activation of NLRP3 and elevated levels of pNF-κB expression [32].

Recent studies have proven that expression of IL-18 and ASC proteins were significantly elevated in the group of patients with diagnosed psoriasis in comparison to healthy controls. The protein level was investigated through the identification of inflammasome component levels in human serum. Moreover, the authors revealed that the expression of IL-18 statistically correlated with that of ASC protein expression levels. Their findings indicate that inflammasome signaling pathway proteins, such as IL-18 and ASC, play a crucial role in inflammatory responses associated with psoriasis pathology. In the future, these proteins could be key biomarkers that help determine the diagnosis of psoriasis in this patient population [35].

Furthermore, recent studies found that BAY 11-7082 is considered an antagonist of causes, not only the activation of NF-kB, but it can also alleviate psoriasis-like dermatitis by inhibiting the NLRP3 inflammasome and the NF-kB pathway [28]. Moreover, MCC950, a recognized NLRP3 inflammasome inhibitor, can significantly alleviate imiquimod-induced psoriasis-like dermatitis [21]. Another group of researchers from China showed that the combination of PlxnB2 and its soluble ligand, CD100, stimulates the production of IL-1β and IL-18 by keratinocytes and activates the NLRP3 inflammasome and the NF-kB pathway during the course of psoriasis [26]. These results confirmed the role of NLRP3 activation in the pathogenesis of this skin disease.

Several triggers were proposed as psoriasis initiator events, including DAMP and other alarming factors, which are released during damage, stress, or necrotic cells after trauma. Extracellular adenosine triphosphate (ATP) is an alarmin that may induce, via purinergic P2 × 7 receptor (P2 × 7R) signaling, the IL-23/IL-17 axis and NF-kB activation. Research shows that both are psoriasis susceptibility pathways. Diaz-Perez et al. confirmed that IL-23 and extracellular ATP may activate P2 × 7R to trigger the development of psoriasis. Moreover, Diaz-Perez et al. found that, the P2 × 7R-induced inflammatory response is mostly dependent on activation of NLRP3 [27,30]. Furthermore, they showed that activation of the NLRP3 inflammasome occurred mostly through neutrophils and not keratinocytes or T cells [30]. After injecting Nlrp3-deficient mice with rIL-23, psoriasiform phenotypes were significantly ameliorated. Thus, NLRP3 inflammasome may contribute to this process. Moreover, research presented that miR-155 may induce psoriasis associated inflammatory responses via NLRP3 inflammasome activation [27].

Interestingly, it was observed that NLRP3 and CARD8 polymorphisms are significantly connected with susceptibility to some inflammatory diseases, including psoriatic juvenile idiopathic arthritis [36] and rheumatoid arthritis [37]. Carlström et al. suggested that the NLRP3 polymorphisms rs4925663, rs35829419, rs10733113, and the CARD8 rs2043211 polymorphism relate to autoinflammatory disease, prompting research into the role of NLRP3 polymorphisms and its components in the pathogenesis of psoriasis [19]. They demonstrated—in a Swedish population—that certain polymorphisms in CARD8 (rs2043211) an NLRP3 (rs10733113) are strongly connected to the increase of psoriasis development risk and in patients with more widespread psoriasis. The role of the NLRP3 in psoriasis susceptibility was also repeated in the Asian population, where, in turn, the authors noted that rs10754557 and rs3806265 in NLRP3 were related to psoriasis, and showed that NLRP3 polymorphisms can be a causative genetic or valuable genetic marker factor in psoriasis development [38].

In addition to the BAY 11–7082, other substances exhibit NLRP3 inhibitory properties, such as cycloastragenol, which is a triterpenoid saponin isolated from various legume species in the genus Astragalus. Cycloastragenol (CAG) significantly reduces imiquimod-triggered NLRP3 inflammasome activation while GSDMD conducts to pyroptosis in proinflammatory macrophages. These substances significantly ameliorate skin inflammation during psoriasis [23].

Moreover, some plants, including *Datura Metel L*. mustard seed and rosmarinic acid (RA), play a protective role against the development of imiquimod-induced psoriasis by inhibiting NLRP3 inflammasome activation [8,26] by cytokines, such as IL-1β, IL-6, IL-8, CCL20, and TNF suppression. Those results could indicate the future direction of inflammasome and psoriasis relation to provide novel therapeutic therapies.

Overall, the role of NLRP3 inflammation in pathogenesis and therapy of pyroptosis was presented at different levels. Nevertheless, there are still some different results, the study of Rabeony et al. found that it is MyD88 and IL-1R1 signaling, not NLRP3 inflammasome, associated with imiquimod-induced skin inflammation in mice [29]. Therefore, further exploration and verification is required to clarify the relevance between NLRP3 inflammasome and psoriasis.

## 3. The NLRP1 Inflammasome (in Psoriasis)

NLRP1 is another inflammasome-forming NLRP family member in humans. NLRP1 was the first described inflammasome, of which molecular mechanisms of activation and the resulting events are still not fully understood [35,39,40]. The activation of NLRP1 is regulated in a very complex way. Mutations of NLRP1 have been observed in patients with most cutaneous inflammatory diseases, demonstrating that NLRP1 plays a particularly major function in the skin and predisposition for chronic inflammatory conditions. Ekman et al. revealed that NLRP1 inflammasome complex genetic variations are related to elevated vulnerability to psoriasis [16]. They analyzed the role and functional effects of several single nucleotide polymorphisms, including rs878329, rs12150220, rs8079034, and rs6502867, in psoriasis. They reported the overtransmission of the NLRP1 rs878329C and rs8079034C genotypes in psoriasis.

Another important finding that confirmed the significant correlation between NLRP1 gene polymorphisms and psoriasis is the demonstration that homozygosity for the rs878329C allele was also correlated with the onset of psoriasis at an earlier age. In addition, researchers revealed that the expression level of NLRP1 mRNA and circulating IL-18 is significantly increased in the peripheral blood of patients suffering from psoriasis. There was a correlation between higher levels of circulating IL-18 and the rs878329C allele. The results of these studies clearly indicate the role of the NLRP1 inflammasome in the development and course of psoriasis [16].

Fenini et al. [41] observed that the NLRP1 inflammasome plays a crucial role in UVB sensing and subsequent IL-1β and IL-18 secretion by human keratinocytes. It was demonstrated that, in myeloid cells, NLRP3 is the most crucial inflammasome, while in keratinocytes, NLRP1 predominates.

Juneblad et al. [42] indicated an association with a genetic polymorphism in an inflammasome-related gene, CARD8-C10X (rs2043211), in patients with PsA. Associations between various phenotypes of PsA and different polymorphisms of the inflammasome genes were also found. Those results indicate the involvement of inflammasome genes in the pathogenesis and disease expression of PsA. However, no association with NLRP1 or NLRP3 was detected.

## 4. The AIM2 Inflammasome (in Psoriasis)

**Figure 2:**
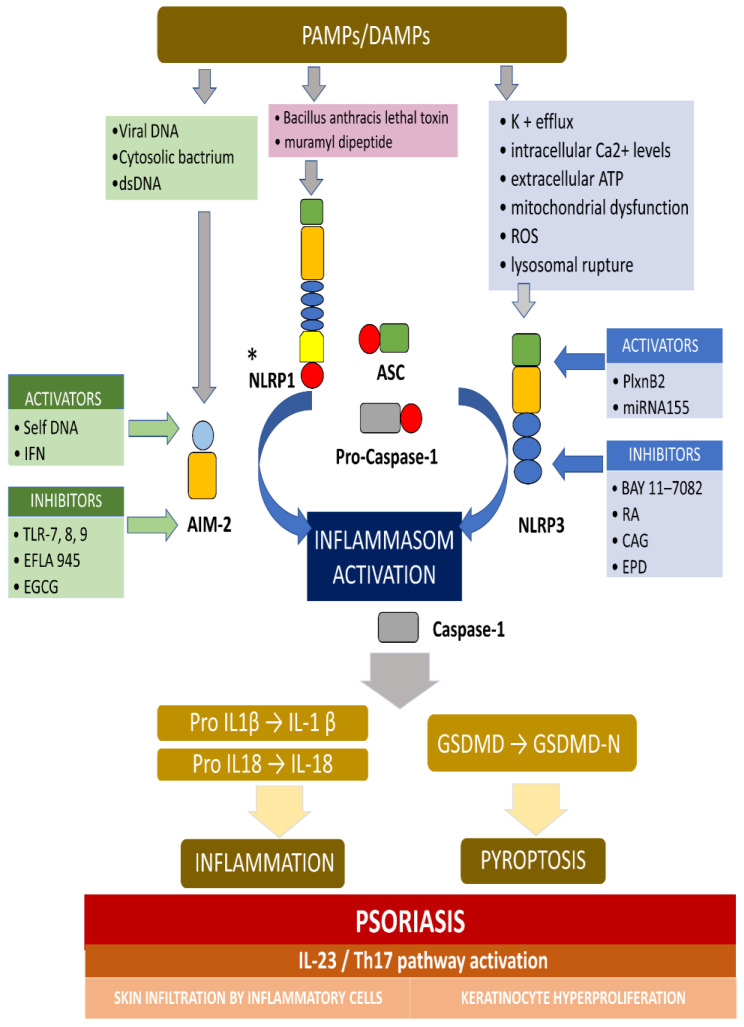
The activation and inhibition of the AIM2 and NLRP3 inflammasome in psoriasis. * Activators and inhibitors are not reported for NLRP1. The AIM2 inflammasome detects cytosolic dsDNA, including DNA viruses and cytosolic bacterium, which causes inflammasome activation. The NLRP3 inflammasome can be activated by a variety of PAMPs and DAMPs, which cause, for example, potassium efflux, extracellular ATP, lysosomal destabilization, intracellular calcium levels, and ROS (reactive oxygen species). When the main components of the inflammasome is connecting and the active inflammasome is formed, it directly recruits and cleaves pro-caspase1 into active caspase-1, which proteolytically activates the pro-inflammatory cytokines IL-1b and IL-18. In addition, the activated inflammasome cleaves gasdermin D (GSDMD) into active N-terminal fragment (GSDMD-N), which drives a lytic type of cell death pyroptosis. In psoriasis, the self-DNA in patients, and IFN in keratinocyte in mouse activate AIM-2 inflammasome, while TLR-7/8/9 (toll-like receptor), EFLA 945 (red vine leaf extract), and EGCG (epigallocatechin-3-gallate) can inhibit the AIM2 inflammasome. In turn, PlxnB2/ligand and miRNA155 activate the NLRP3 inflammasome, while BAY 11-7082, rosmarinic acid (RA), cycloastragenol (CAG) and EPD (the effective part of *Datura metel L*.) inhibit this inflammasome. The active IL-1b and IL-18 activates IL23/Th17 pathway, inducing a numerous of inflammatory cytokines and chemokines. Different kinds of immune cells infiltrating into the skin and, finally, causing hyperproliferation in the epidermis, was observed.

AIM2 is a member of the cytosolic innate immune receptor, which detects the presence of cytosolic double-stranded DNA (dsDNA) from intracellular bacteria, viruses, and self-DNA. The AIM2 recognition of dsDNA results in the assembly of inflammasome and the secretion of the proinflammatory IL-1β and IL-18. AIM2 plays a crucial role for the NLRP3 inflammasome in host defense against various pathogens and may also contribute to autoimmune or autoinflammatory disorders. It was revealed that, in normal conditions, AIM2 is expressed solely in melanocyte and Langerhans cells in an ordinary, healthy epidermis, while AIM2 expression is meaningfully elevated in keratinocytes in inflammatory conditions, including atopic dermatitis, allergic contact dermatitis, and psoriasis [20]. Dombrowski et al. presented that cytosolic DNA activated AIM2 inflammasomes, resulting in the production of proinflammatory cytokines, only in keratinocytes, in psoriatic lesions (not in healthy lesions) [20]. Figure 2 presents the activation and inhibition of AIM2 and the NLRP3 inflammasome in psoriasis.

However, the source of cytosolic DNA in psoriatic keratinocytes is not clarified, the theory about extracellular DNA comes from dying cells, and may be recognized by cytosolic AIM2 that promotes sterile inflammatory skin diseases [43]. The extracellular self-DNA, which is normally removed via deoxyribonuclease (DNase), may aggregate with the antimicrobial cathelicidin peptide LL-37 and may activate psoriasis [44]. Dombrowski et al. investigated the role of LL-37 in DNA-triggered inflammation and they hypothesized that interaction LL-37, strongly expressed in skin in psoriasis and cytosolic DNA interactions, might contribute to AIM2-dependent inflammasome activation [43]. However, as it was revealed, the LL-37-DNA complex internalized into the cytosol loses its ability to activate the AIM2 inflammasome. The results of this study show that the cathelicidin LL-37 peptide acts as a natural inhibitor of the AIM2 inflammasome assembly. As later observations showed, activation of the AIM2 inflammasome occurs in untreated psoriatic lesions, despite increased expression of LL-37. This suggests that untreated psoriasis levels of LL-37 may be insufficient at inhibiting the activity of the AIM2 inflammasome. On the other hand, it was shown that treatment of patients with psoriasis, with UVB radiation, which enhances cutaneous vitamin D synthesis, or with local vitamin D analogues, strongly induces cathelicidin expression in damaged skin, alleviating skin inflammation [45,46]. The findings from Dombrowski et al. not only provide a direct explanation of the success of the aforementioned therapies (UVB, vitamin D), but also suggest that the cathelicidin LL-37 peptide may be a new therapeutic target for anti-psoriatic therapies, affecting inflammasomes, including AIM2 [40].

Undoubtedly, the pro-inflammatory cytokine IL-1β plays a crucial role in the pathogenesis of many inflammatory diseases of the human skin, including psoriasis, but the keratinocytes of murine models, with many studies, do not express IL-1β, only cytokine IL-18, which can be cleaved to its active form by inflammasomes in these cells [47]. Cathelicidin LL-37 can interact with DNA and is highly expressed in psoriasis; it is hypothesized that LL-37–DNA interactions may contribute to activation of AIM2 dependent inflammasome [48]. The cathelicidin mRNA expression in lesional skin in psoriasis patients was, on average, 19-fold higher compared to healthy donors (*p* < 0.01), according to Dobrowski et al. [20].

Dermal cathelicidin LL-37 binds self-DNA and triggers cutaneous inflammation by activation of dermal plasmacytoid dendritic cells, while epidermal LL-37 joins with cytosolic DNA in keratinocytes and blocks its pro-inflammatory activity [24,25]. This may explain why vitamin D3, a strong inducer of cathelicidin expression in keratinocytes and monocytes, is effective for psoriasis and reduces inflammation in psoriatic lesions [23].

Toll-like receptor (TLR) superfamily members are key transmembrane proteins that play a crucial role in both the innate and adaptive immune responses contributing to the pathogenesis of psoriasis. In murine models, administration of IL-23 causes clinical symptoms associated with psoriasis, such as hyperproliferation of keratinocytes and thickening of the epidermis with mononuclear cell infiltration [49,50,51]. Treatment with TLR-7, -8, and -9 antagonists in these mice was shown to reduce both psoriasis-related skin lesions, inhibit the dermal expression of NLRP3 and AIM2, and decrease the secretion of Th1 and Th17 cytokines in the skin and serum. Reaffirming the role of inflammasomes as a potential therapeutic target in the treatment of psoriasis [9], as with the NLRP3 inflammasome, researchers focused on finding substances that would silence or completely inhibit AIM-2 inflammasome activation in psoriasis. In 2020, Ching et al. demonstrated in IMQ-induced psoriasis models that red vine leaf extract (EFLA 945) may interfere with the activation of AIM2 and other inflammasomes; therefore, it has obvious potential in the treatment of psoriasis [24].

In contrast, IL-18 receptor knockout mice treated with the Aldara–product, consisting mainly of isostearic acid and IMQ, showed a greater, thicker epidermis than that seen in normal controls. The authors attributed the described phenomenon to isostearic acid, which is the main component of NLRP1 activation in a murine model, indicating that Aldara might stimulate psoriasis-like phenotypes in different immune pathways, requiring both inflammasome and IMQ induced response [25]. Aldara also induces psoriasis-like lesions when applied to naive murine skin, and as such, is used as a mouse model for psoriasis [25].

Another substance that was shown to inhibit AIM2-induced inflammatory cytokines in human epidermal keratinocytes is epigallocatechin-3-gallate (EGCG), a component extracted from green tea. EGCG reduced the level of the IFN-γ-induced priming signal via downregulation of pro-IL-1b and pro-capspase-1 in human epidermal keratinocytes, neonatal cells [23]. Recently, cytosolic DNA was identified as a danger signal that activates inflammasomes containing the DNA sensor AIM2.

## 5. Conclusions

In this systemic review, we highlighted, and attempted to associate, major scientific breakthroughs that revealed clues to the pathophysiology of psoriasis. We aimed to provide order to the various pathophysiological mechanism-involved inflammasomes that contribute to the development of the autoimmune background of psoriasis. The exact role of inflammasomes and their components that lead to the initiation of the psoriasis cascade remains unknown, but the identification of early triggers of the immune system may provide novel, promising therapeutic targets for the prevention and control of psoriasis. This review summarizes and discusses recent studies that explore the regulatory roles of inflammasomes during psoriasis, and provides insight into the development of novel therapeutics for psoriasis, by targeting protein components of canonical and non-canonical inflammasomes.

## Figures and Tables

**Table 1:** The inflammasome in psoriasis.

Author	Inflammasome	Effector Signal	Cell Type	Mouse/Human	Main Finding
Dombrowski et al. [20]	AIM2	IL-1β	Keratinocytes	Human	Cytosolic DNA can trigger AIM2 inflammasome IL-1β activation in psoriasis; cathelicidin LL-37 interfered with DNA-sensing inflammasomes.
Ekman et al. [16]	NLRP1	IL-1β, IL-18	n/a	Human	NLRP1 inflammasome complex genetic variations are related to elevated vulnerability to psoriasis.
Deng et al. [21]	NLRP3	IL-1β	Dendritic cells, Neutrophils, T lymphocytes	Mouse	CAG significantly reduced imiquimod-triggered NLRP3 inflammasome activation and gasdermin D (GSDMD)-mediated pyroptosis.
Su et al. [22]	NLRP3	IL-1β, caspase 1	Keratinocytes	Human	The expression of NLRP3 in psoriatic samples was 3.5 to 4.3 times higher than the expression of NLRP3 in normal skin biopsy samples; NLRP3 expression is upregulated in psoriasis and is associated with concomitant accumulation of IL-1b and caspase 1.
Yun et al. [23]	AIM2	IL-1β	HEKn	Human	EGCG reduces AIM2-induced IL-1b secretion by suppressing IL 1b-mediated priming and poly-induced ASC oligomerization.
Chung et al. [24]	AIM2	IL-1β, IL-18	Macrophages	Human	EFLA 945 attenuates IMQ-induced psoriasis-related proinflammatory responses;
Jiang et al. [9]	AIM2	IL-1β, IL-18	n/a	Mouse	the inflammasome expression components NLRP3 and AIM2 are reduced by antagonist treatment.
Walter et al. [25]	NLRP1	IL-1β, IL-18	Neutrophils, Keratinocytes	Mouse	NLRP1 inflammasome activation is promoted by isostearic acid in cultured keratinocytes.
Zhang et al. [26]	NLRP3	IL-1β	Keratinocytes	Human	NLRP3 inflammasome is activated by CD100 in keratinocytes through binding to PLXNB2.
Luo et al. [27]	NLRP3	IL-1β, IL-18	Keratinocytes	Mouse	miR-155 activates the NLRP3 inflammasome; miR-155 does not impact the TLR4/NF-kB signaling pathway.
Yang et al. [26]	NLRP3	IL-1β	Keratinocytes	Mouse	EPD inhibits the production of imiquimod-induced inflammatory cytokines
Irrera et al. [28]	NLRP3	IL-1β, IL-18	Keratinocytes	Mouse	BAY 11-7082 alleviates the NLRP3 and dual NF-kB.
Rabeony et al. [29]	NLRP3	n/a	n/a	Mouse	IMQ-induced skin inflammation is independent on the NLRP3 inflammasome.
Diaz–Perez et al. [30]	NLRP3	IL-1β	Macrophages, Granulocytes, Neutrophils	Mouse	P2 × 7R-induced inflammation is largely dependent on the IL-1b/NLRP3 inflammasome pathway and neutrophils.
Shi et al. [31]	NLRP3	IL-1β	Keratinocytes	Mouse	Caspase-11 and caspase-1 can cleave GSDMD, revealing a pyroptosis-inducing fragment
Yang et al. [32]	NLRP3	IL-1β	Keratinocytes	Mouse	EPD exhibited a protective effect on an imiquimod-induced psoriasis mice model by inhibiting the inflammatory response, which might be ascribed to the inhibition of the TLR7/8–MyD88–NF-κB–NLRP3 inflammasome pathway
Forouzandeh et al. [32]	NLRP3	ASC, IL-1β, IL-18	Keratinocytes	Human	ASC and IL-18 play a significant role in the inflammatory response associated with the pathology of psoriasis. These inflammasome proteins appear to be key biomarkers in determining diagnoses in this patient population.

n/a: not available.

## Data Availability

No new data were created or analyzed in this study. Data sharing is not applicable to this article.
